# Wheat growth, applied water use efficiency and flag leaf metabolome under continuous and pulsed deficit irrigation

**DOI:** 10.1038/s41598-020-66812-1

**Published:** 2020-06-22

**Authors:** Jana Stallmann, Rabea Schweiger, Caroline A. A. Pons, Caroline Müller

**Affiliations:** Department of Chemical Ecology, Bielefeld University, Universitätsstr. 25, 33615 Bielefeld, Germany

**Keywords:** Plant stress responses, Ecology

## Abstract

The intensity and frequency of precipitation events are predicted to change over the coming decades. For many areas, longer periods without rainfall are expected. We investigated the importance of irrigation frequency under water deficit conditions for growth, physiology and chemistry of wheat (*Triticum aestivum*). Drought-stressed plants received 40% of the water provided for control plants and were either watered every other day (continuous drought, cd) or every eight days (pulsed drought, pd). Maximum quantum yield of photosystem II (F_v_/F_m_), aboveground biomass, applied water use efficiency (WUE_apl_) and the flag leaf metabolome were assessed twice during development. F_v_/F_m_ was not affected by irrigation. Drought-exposed plants produced less biomass, but had higher WUE_apl_ than control plants. More metabolic features responded to the pd compared to the cd treatment and more features were increased than decreased in pool size in flag leaves. Salicylic acid glucoside was generally decreased under drought. In pd plants, two benzoxazinoid glucosides were enhanced at the first time point and concentrations of several flavonoid glycosides were modulated. This study extends our knowledge about drought effects on wheat; it highlights that the frequency of watering determines how plant growth, physiology and metabolism are affected by drought.

## Introduction

Due to global climate change, the intensity and frequency of extreme climate events like drought periods and heat waves are predicted to increase^1^. Longer drought periods, often followed by floods, lead to irregular water availability for plants. About 80% of the world’s agriculturally used area is rain-fed^2^, but a large part of this area will need supplemental irrigation in the future, implicating new challenges for agricultural practice. Many laboratory studies examined the impacts of continuous drought on plant physiology and chemistry^3,4^. However, for the predicted climate change scenarios, it is important to not only focus on the amount of water provided but also on the frequency of water availability^1^, which has rarely been investigated (but see^5^). Especially studies on the effects of continuous vs. pulsed water availability on the phytometabolome are lacking but may reveal a deeper understanding of plant responses to climate change events important for crop breeding.

Drought stress does not only result in a reduced vegetative shoot biomass^6^ but also reduces grain yields of many crops^7^. Shoot fresh biomass of *Pelargonium* x *hortorum* (Geraniaceae) was reduced particularly under infrequent compared to frequent deficit irrigation, indicating that both the total water volume and the frequency of watering are critical for plant growth^5^. The intrinsic plant water use efficiency (WUE_i_), i. e., the CO_2_ assimilation rate divided by the transpiration rate or stomatal conductance, often increases under mild water scarcity^8^. This might be due to a reduced stomatal conductance, restricting the transpirational water loss earlier and more effectively than lowering photosynthesis^8^. The applied water use efficiency (WUE_apl_) describes how efficiently water is used for plant biomass production and is thus highly relevant for water-limited agricultural systems.

Together with physiological changes, several primary metabolites accumulate under drought^9^, leading to a decreased plant osmotic potential that facilitates the uptake of water from the soil^10^. In particular, the amino acid proline shows higher concentrations in plants under drought^11^ and functions as an osmolyte, stabilises membranes and proteins and detoxifies reactive oxygen species^12^. Furthermore, sugars and sugar alcohols are usually higher concentrated in leaves of drought-stressed compared to well-watered plants^13,14^. Likewise, in many plant species concentrations of specialised (=secondary) metabolites such as pyrrolizidine alkaloids^15^ or terpenoids^16^ increase under drought, but also decreased concentrations of specialised compounds were found^17,18^. Moreover, the metabolic responses to drought can differ between plant parts^6^ and may depend on the duration of drought^16^. Apart from changes in target primary and specialised metabolites, little is known about the magnitude and direction of overall metabolic changes in response to continuous and pulsed drought stress. However, for maize plants subjected to salt and drought stress, evidence was found for a relationship between physiological and metabolic changes^19^. (Eco-)metabolomics is an effective approach to investigate plant responses to diverse stresses^20^, whereby untargeted metabolic fingerprinting can reveal shifts in large parts of the phytometabolome^21^.

As one of the world’s most important crop plant species, common wheat (*Triticum aestivum*) is cultivated in many temperate regions. Global wheat yields are steadily increasing^22^, but the rate of yield increase will probably not be sufficient to cover the rising demand imposed by the growing human world population^22^. Drought stress does not only influence wheat yield^23^ but also leads to changes in foliar primary metabolites, with the effects differing between cultivars of different drought tolerance^24^. Next to these well-investigated primary metabolites, specialised metabolites of wheat may respond to drought. As characteristic specialised metabolites, wheat synthesises several shikimic acid-derived compounds. For example, benzoxazinoids (BXDs) and their glucosides are derived from tryptophan, have insecticidal^25^ as well as allelopathic properties^26^ and act both directly as defence compounds or indirectly, being involved in signalling for callose deposition upon attack^27^. Drought stress leads to increased concentrations of BXDs in young wheat seedlings^28^. Moreover, diverse phenylalanine-derived specialised metabolites occur in wheat^29^. Flavonoids and their glycosides are probably particularly relevant under drought stress, as they may help the plants to cope with secondary oxidative stress. Indeed, the important role of flavonoids and their glycosides during drought and oxidative stress was highlighted in *Arabidopsis thaliana* (Brassicaceae)^30^. In contrast to many other plant species containing *O*-glycosylated flavonoids, cereals including wheat also synthesise *C*-glycosylated flavonoids. *C*-glycosyl flavones have been shown to increase in response to abiotic stress like nitrogen limitation^31^ and low temperature^32^ in wheat leaves. However, the direction and magnitude of effects of drought stress at different irrigation frequencies on the phytometabolome and particular pathways within the specialised metabolism of wheat have, to our knowledge, not been studied until now.

In the present study, we aimed to investigate the effects of different drought regimes on growth, physiology and flag leaf chemistry of wheat. Therefore, we subjected wheat plants to control conditions (well-watered) or to continuous drought stress or pulsed drought stress, with plants of both stress treatments receiving the same total amount of water. At two time points during the growth period, we determined the maximum quantum yield of photosystem II, the aboveground biomass and WUE_apl_. At the second time point, we differentiated between investment in vegetative and generative aboveground biomass and WUE_apl_. Additionally, we analysed the (semi-)polar metabolome of flag leaves harvested at both time points by untargeted metabolic fingerprinting followed by identification of several characteristic specialised metabolites. We predicted that drought stress, especially at low irrigation frequency, results in a lower aboveground dry mass. Furthermore, we hypothesised that WUE_apl_ increases under drought, most notably in continuously drought-stressed plants. Moreover, we expected that many specialised metabolites increase in concentrations under drought. We assumed that these metabolic responses have a higher magnitude at a lower irrigation frequency.

## Results

**Figure 1:**
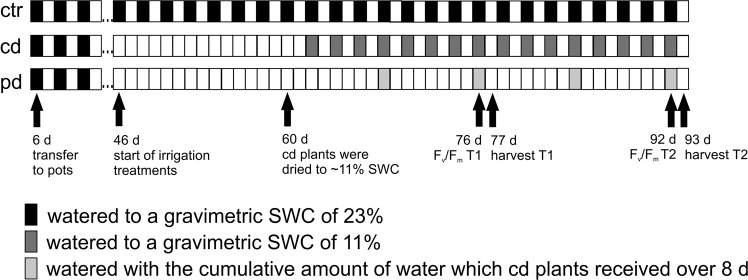
Schematic overview of the experimental design. All plants were potted 6 days after sowing and were watered every other day (filled boxes) until the beginning of irrigation treatments (ctr, well-watered control plants; cd, continuously drought-stressed plants; pd, plants subjected to pulsed drought). SWC = soil water content.

**Figure 2:**
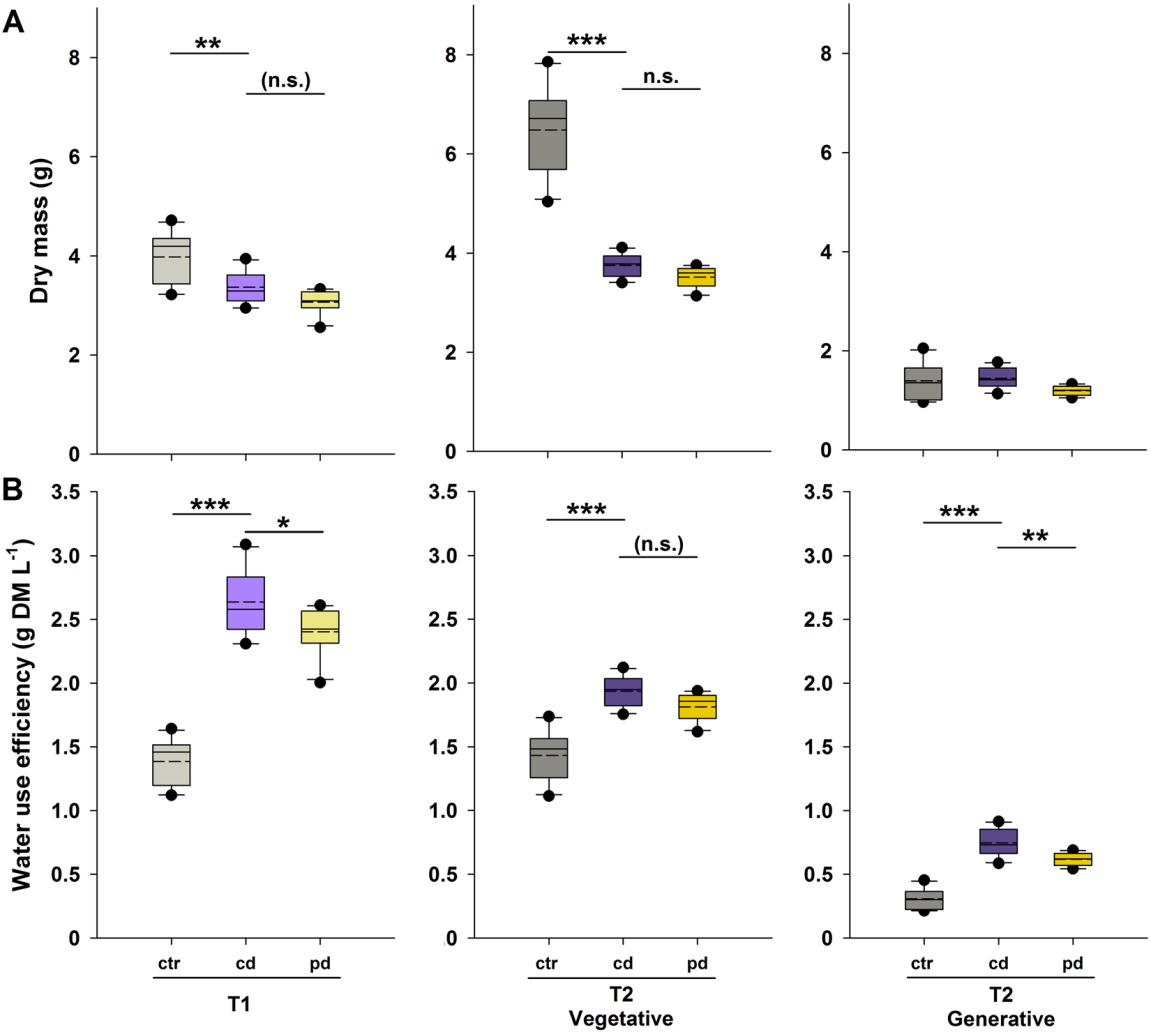
(**A**) Aboveground dry mass and (**B**) applied water use efficiency of wheat plants subjected to well-watering (ctr), continuous (cd) and pulsed (pd) drought, harvested at two time points (T1 = 77 d and T2 = 93 d after sowing). The applied water use efficiency was calculated for each pot as the ratio of aboveground plant dry mass to the cumulative amount of water received until harvest. At T2, values are given for vegetative (leaves and stems) and generative plant parts (ears). The boxes represent the interquartile ranges, whiskers extend to the 10% and 90% percentiles, respectively; solid lines show the medians, dashed lines the means. Outliers are shown as circles; when there was a significant effect of irrigation treatment (Table 2), manual contrasts between selected groups were calculated and *p* values are given; ***p < 0.001; **p < 0.01; *p < 0.05; (n.s.) marginally significant (*p* < 0.1); n.s. not significant; *n* = 10.

Wheat plants were grown in a greenhouse and pots assigned to one of three irrigation treatments, a well-watered control (ctr), continuously drought-stressed (cd) and pulsed drought-stressed (pd) plants (Fig. 1). On day 76 and 92 after sowing (30 and 46 after the start of the irrigation treatments), the maximum quantum yield of photosystem II (F_v_/F_m_) was determined on the flag leaves. F_v_/F_m_ did not differ significantly between plants of different irrigation treatments and time points (Table 1). Plants were harvested at day 77 (T1) and 93 (T2) after sowing. At T1, the total aboveground plant dry mass was influenced by the irrigation treatment, being significantly lower in cd compared to ctr plants and slightly higher (marginal significance, *p* < 0.1) in cd compared to pd plants (Fig. 2A, Table 2). At T2, ctr plants had produced the highest vegetative dry mass. Plants of the pd treatment were smallest, but their vegetative dry mass did not differ significantly from that of cd plants (Fig. 2A). In contrast, the generative dry mass was not influenced by the irrigation treatment (Fig. 2A, Table 2). The harvest index (ratio of grain yield to total aboveground dry mass) was significantly lower for ctr compared to drought-stressed plants and significantly higher for cd compared to pd plants (data not shown). The WUE_apl_ was significantly influenced by the irrigation treatment, both for vegetative (T1, T2) and generative (T2) plant parts, whereby ctr plants showed the lowest WUE_apl_ and cd plants had higher WUE_apl_ than pd plants (Fig. 2B, Table 2).

**Table 1:** Results of a generalised linear model (error family: Gamma, link function: inverse) for treatment effects on the maximum quantum yield of photosystem II (F_v_/F_m_) of wheat flag leaves. The effects of the factors irrigation treatment, time point and their interaction were tested; *n* = 10.

Stat.	Null model	Irrigation treatment (IT)	Time point (TP)	IT *x* TP
df	59	57	56	54
dev.	0.2	0.1	0.1	0.1
*p*		0.949	0.979	0.199

**Table 2:** Results of linear models for treatment effects on wheat traits.

	Stat.	Null model	Irrigation treatment	ctr *vs*. cd	cd *vs*. pd
Total dry mass T1	df	29	27		
	dev.	8.16	3.85		
	*p (F)*		**<0.001** (15.13)	**0.002**	0.088
Vegetative dry mass T2	df	29	27		
	dev.	62.66	8.20		
	*p (F)*		**<0.001** (89.63)	**<0.001**	0.341
Generative dry mass T2	df	29	27		
	dev.	1.98	1.63		
	*p (F)*		0.072 (2.91)		
Total WUE_apl_T1	df	29	27		
	dev.	10.04	1.18		
	*p (F)*		**<0.001** (101.33)	**<0.001**	**0.019**
Vegetative WUE_apl_T2	df	29	27		
	dev.	1.96	0.59		
	*p (F)*		**<0.001** (31.64)	**<0.001**	0.073
Generative WUE_apl_T2	df	29	27		
	dev.	1.20	0.18		
	*p (F)*		**<0.001** (74.99)	**<0.001**	**0.002**

The effects of the factor irrigation treatment on vegetative and generative aboveground dry mass and applied water use efficiency (WUE_apl_) were tested for both time points (T1, T2) separately. Manual contrasts for comparison of ctr *vs*. cd and cd *vs*.pd were calculated, if there was a significant effects of the irrigation treatment and *p* values of the contrasts were corrected according to Holm within each model; significant *p* values are highlighted in bold; *n* = 10.

**Figure 3:**
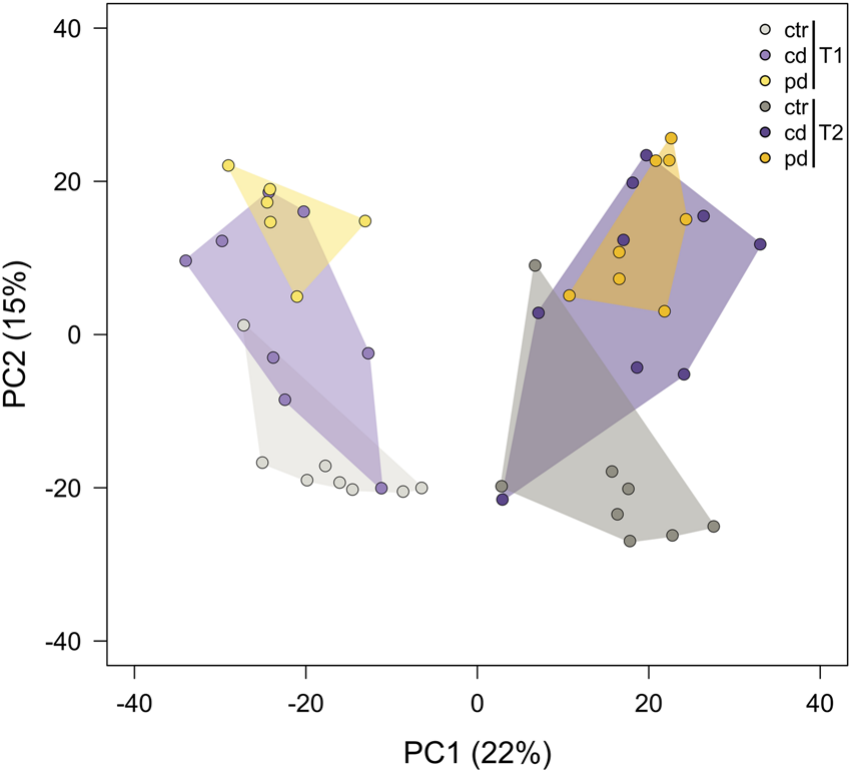
Principal component analysis showing the metabolic composition (including 1,958 metabolic features) of wheat flag leaves. Plants were subjected to different irrigation regimes (ctr, well-watered control; cd, continuously drought-stressed plants; pd, plants subjected to pulsed drought) and harvested at two time points (T1 = 77 d and T2 = 93 d after sowing). Symbols show scores of the 6-9 biological replicates. Data were autoscaled and zeros replaced by random small numbers. Percent total variances explained by the principal components are shown in brackets and groups are surrounded by convex hulls.

**Figure 4:**
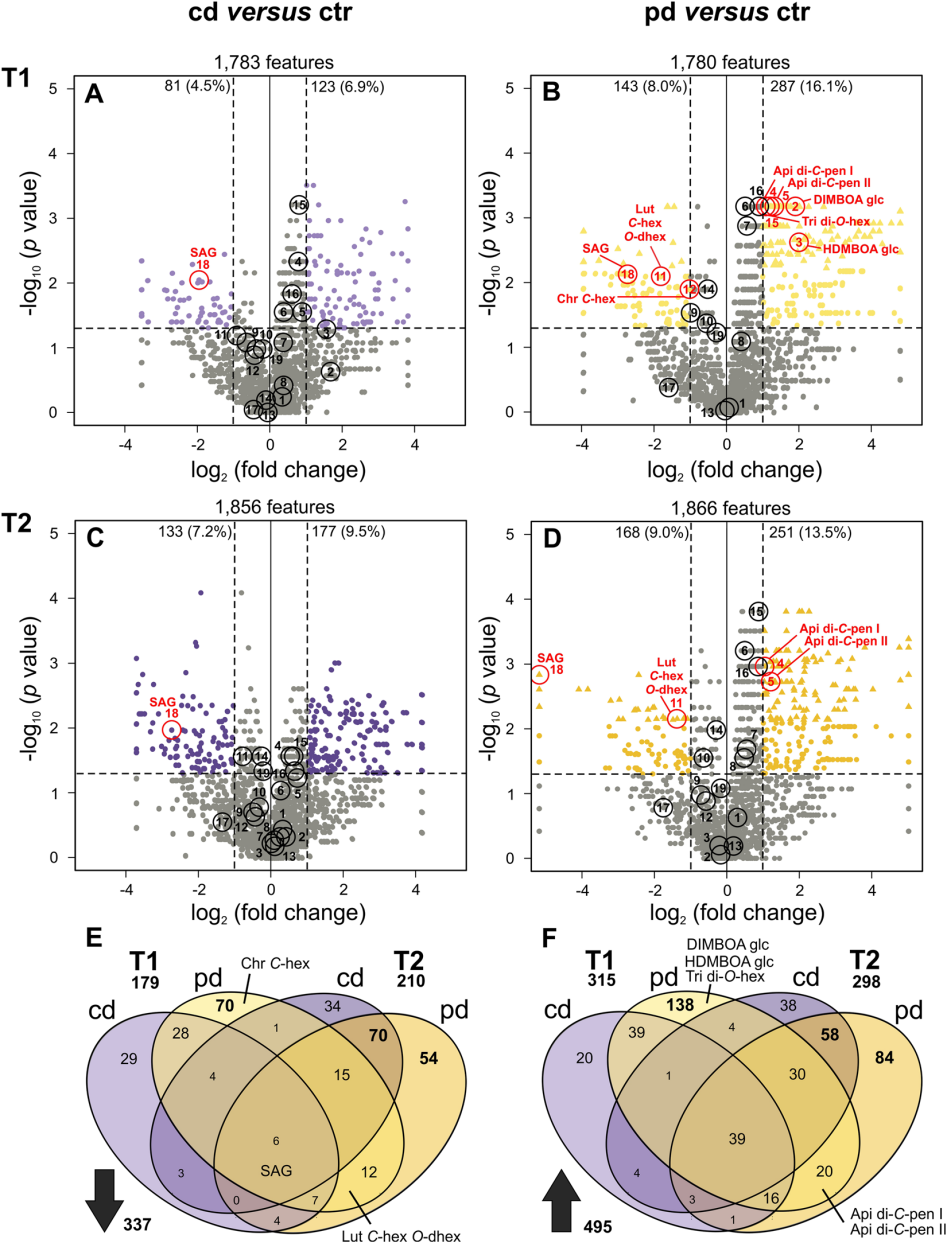
Metabolic responses of wheat flag leaves to drought stress. (**A**–**D)**: Volcano plots showing metabolic features in wheat flag leaves subjected to continuous (cd: **A**, **C**) and pulsed (pd: **B**, **D**) drought stress in comparison to the control (ctr) group, 77 d (T1: **A**, **B**) and 93 d (T2: **C**, **D**) after sowing. Horizontal dashed lines represent a *p* value of 0.05. For each metabolic feature, the negative log_10_ of the *p* value (Mann-Whitney *U*-test) is plotted against the log_2_ of the mean fold change. Coloured circles represent features with an unadjusted *p* value of <0.05 and a fold change of <0.5 (decreased in pool size) or >2 (increased in pool size). The number and percentage of features that are decreased or increased is given for each comparison. Coloured triangles represent features with a *p* value of <0.05 after Benjamini and Hochberg^65^ correction for multiple testing. Open circles mark putatively identified metabolites, with the labels corresponding to the metabolite numbers in Table 3. For metabolites that were modulated by drought, the circles are highlighted in red and metabolite abbreviations are given in addition. (**E**,** F)** Venn diagrams depicting the overlapping plant responses to continuous *versus* pulsed drought stress at the different time points, with numbers of metabolic features being decreased (**E**) or increased (**F**) in pool size according to *p* values and fold changes (see above); *n* = 6–9.

At T1 and T2, flag leaves were harvested from separate plants for metabolomics analyses. In total, 1,958 leaf metabolic features were retained in the dataset and included in a principle component analysis (PCA) (Fig. 3). The phytometabolomes of flag leaves pronouncedly differed between the time points of harvest as depicted by the separation of T1 and T2 along PC1. Moreover, phytometabolomes of ctr plants and pd plants were separated along PC2. For pairwise comparisons of the drought groups to the ctr group, features not occurring in both groups were excluded, resulting in 1,780 to 1,866 features (Fig. 4). Up to 9.0% of these features were decreased in pool size by one drought treatment (pd T2, Fig. 4D). In all treatment groups, the percentages of metabolic features that were increased in pool sizes were higher than the percentages of metabolic features that were decreased. Up to 16.1% of the features were increased in pool size by one drought treatment (pd T1, Fig. 4B). Generally, pulsed drought had more pronounced effects on the foliar metabolome than continuous drought; at both time points, the percentages of features that were modulated by the deficit irrigation treatment were higher in pd than in cd plants.

Some of the metabolic features that were modulated [i.e., fold change <0.5 (<−1 on log_2_ scale) or > 2 (>1 on log_2_ scale) and *p* < 0.05] under drought were commonly de- or increased under both irrigation frequencies, whereas others specifically responded only to one of the drought treatments (Fig. 4E,F). Only 2% (decreased) and 8% (increased) of the modulated features were commonly modulated by both drought stress treatments at both time points. At the first time point, 25% (decrease) and 30% (increase) of the modulated features commonly responded to both drought treatments, whereas the majority of the features specifically responded to only one of the treatments. Many more features responded specifically to the pd (i.e, 55% decreased, 61% increased) than to the cd treatment (i.e., 20% decreased, 9% increased). At the second time point, more features were commonly modulated by both drought treatments (43% decreased, 44% increased) compared to T1, whereas again the majority of responses was specific especially for the pd treatment (37% decreased, 41% increased; cd treatment: 20% decreased, 15% increased).

**Figure 5:**
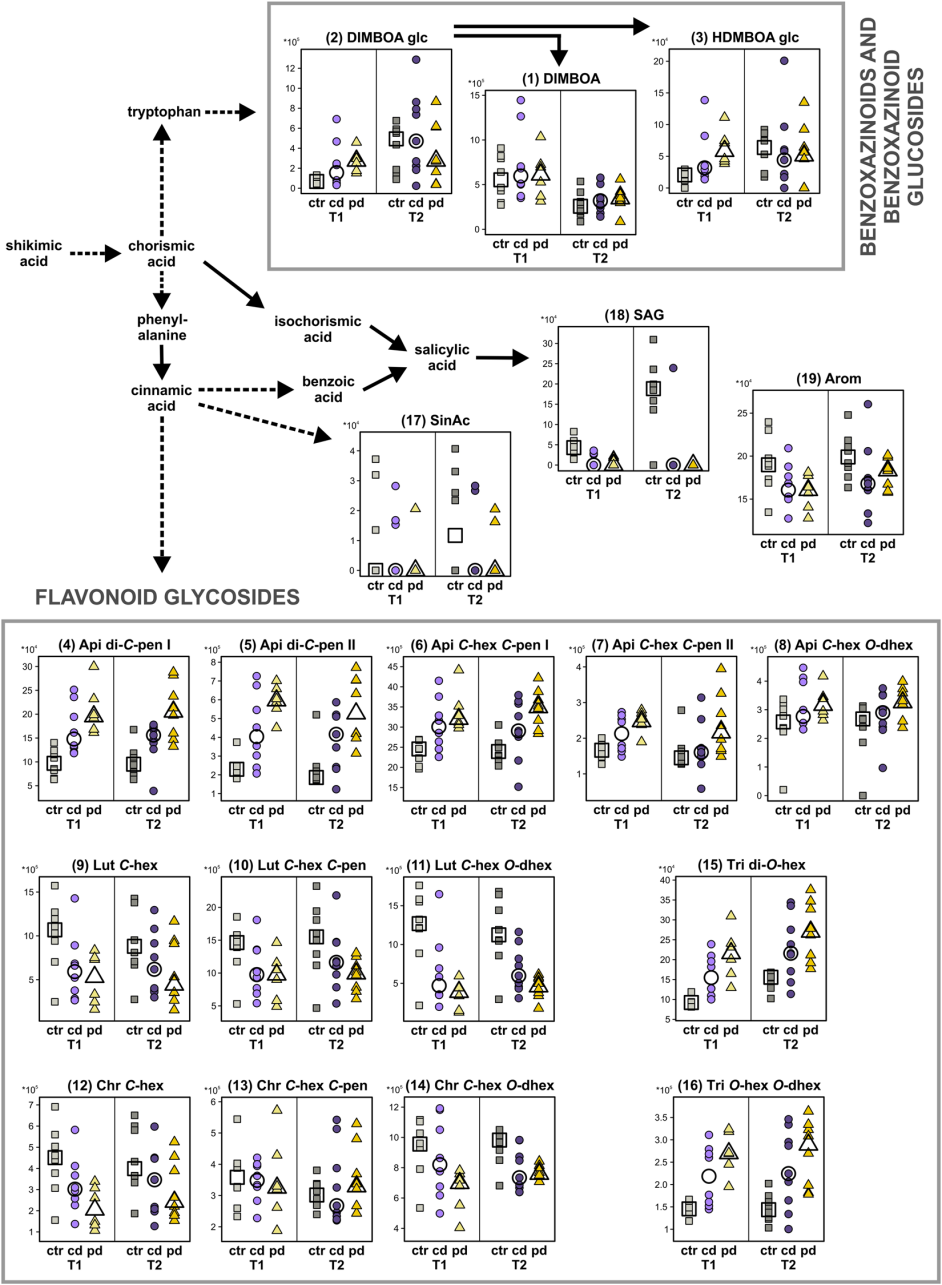
Pathway map of putatively identified metabolites from the shikimic acid pathway in wheat flag leaves. Stripcharts show the concentrations of the corresponding metabolic features in well-watered (ctr), continuously drought-stressed (cd) and pulsed drought-stressed (pd) samples at both time points (T1 and T2). Medians are depicted as large open symbols; *n* = 6–9. Compound numbers and abbreviations refer to Table 3.

Nineteen metabolites could be putatively identified, including one BXD (DIMBOA) and two BXD glucosides (DIMBOA glucoside, HDMBOA glucoside), glycosides of flavonoids, sinapic acid and salicylic acid glucoside (Table 3). All metabolites are part of or derived from the shikimic acid pathway (Fig. 5). The group of flavonoid glycosides was highly diverse (Table 3). Mono- as well as di-glycosides of four flavones (i. e., apigenin, luteolin, chrysoeriol, tricin) were found, with *C*- as well as *O*-glycosidic bonds and different sugars (pen = pentose, hex = hexose, dhex = deoxyhexose) attached. Salicylic acid glucoside was commonly decreased both by cd and pd at both time points (Fig. 4). In contrast, several specialised metabolites were specifically modulated by pd but not cd, either at T1 only or at both time points. Some flavonoid glycosides were decreased (luteolin *C*-hex *O*-dhex) or increased (two isomers of apigenin di-*C*-pen) under pd at both time points. In contrast, the BXD glucosides as well as a tricin di-*O*-hex were specifically increased in pd plants at the first time point only. Thus, leaf metabolic responses to drought were not only highly specific for certain parts of the metabolic pathway (Fig. 5), but also for the irrigation frequency treatment and the time point of harvest.

**Table 3:** (Putatively) identified metabolites found in wheat flag leaves.

Compound class	No.	(Partial) identification	Molecular and structural formulas	RT (min)	Ions MS mode	Fragments MS/MS mode
BXDs and BXD glucosides	**1**	2,4-dihydroxy-7-methoxy-1,4-benzoxazin-3-one DIMBOA (DIMBOA)*	C_9_H_9_NO_5_	8.68	210.041 [M-H]^−^**164.035 [C**_**8**_**H**_**6**_**NO**_**3**_**]**^−^	→21.6 eV: **149** [C_7_H_3_NO_3_]^−^, 121 [C_6_H_3_NO_2_]^−^
	**2**	2-b-D-glucopyranosyloxy-4-hydroxy-7-methoxy-1,4-benzoxazin-3-one DIMBOA glucoside (DIMBOA glc)*	C_15_H_19_NO_10_	7.85	418.100 [M + HCOOH-H]^−^**372.094 [M-H]**^−^	→26.8 eV: 192 [C_9_H_6_NO_4_]^−^, 164 [C_8_H_6_NO_3_]^−^, **149 [C**_**7**_**H**_**3**_**NO**_**3**_**]**^−^
	**3**	2-b-D-glucopyranosyloxy-4,7-dimethoxy-1,4-benzoxazin-3-one HDMBOA glucoside (HDMBOA glc)*	C_16_H_21_NO_10_	10.89	**432.115 [M** + **HCOOH-H]**^−^	→28.3 eV: **194 [C**_**9**_**H**_**8**_**NO**_**4**_**]**^−^, 164 [C_8_H_6_NO_3_]^−^, 149 [C_7_H_3_NO_3_]
Flavonoid glycosides	**4**	**apigenin 6,8-di-*C*-pen**, isomer I **(Api di-*C*-pen I)**	C_25_H_26_O_13_	11.03	**533.131 [M-H]**^−^	→30.7 eV: 515 [(M-H)−18]^−^, **473 [(M-H)−60]**^−^, **443 [(M-H)−90]**^−^, 425 [(M-H)−90-18]^−^, 383 [Ag+113]^−^, 353 [Ag+83]^−^
	**5**	**apigenin 6,8-di-*C*-pen**, isomer II **(Api di-*C*-pen II)**	C_25_H_26_O_13_	11.45	**533.131 [M-H]**^−^	→30.7 eV: 515 [(M-H)−18]^−^, **473 [(M-H)−60]**^−^, **443 [(M-H)−90]**^−^, 425 [(M-H)−90-18]^−^, 383 [Ag+113]^−^, 353 [Ag+83]^−^
	**6**	**apigenin** ***C*-hex**, ***C*-pen**, isomer I **(Api** ***C*-hex** ***C*-pen I)**	C_26_H_28_O_14_	9.62	**563.142 [M-H]**^−^	→31.3 eV: 545 [(M-H)−18]^−^, 503 [(M-H)−60]^−^, **473 [(M-H)−90]**^−^, **443 [(M-H)−120]**^−^, 383 [Ag+113]^−^, 353 [Ag+83]^−^
	**7**	**apigenin** ***C*-hex**, ***C*-pen**, isomer II **(Api** ***C*-hex** ***C*-pen II)**	C_26_H_28_O_14_	10.52	**563.142 [M-H]**^−^	→31.3 eV: 545 [(M-H)−18]^−^, 503 [(M-H)−60]^−^, **473 [(M-H)−90]**^−^, **443 [(M-H)−120]**^−^, 383 [Ag+113]^−^, 353 [Ag+83]^−^
	**8**	**apigenin** ***C*-hex, 2”-*O*-dhex (Api C-hex** ***O*-dhex)**	C_27_H_30_O_14_	11.23	**577.157 [M-H]**^−^	→31.5 eV: 457 [(M-H)−120]^−^, **413 [(M-H)−164]**^−^, 341 [Ag+71]^−^, 323 [Ag+71-18]^−^, 311 [Ag+41]^−^, **293 [Ag** + **41-18]**^−^
	**9**	**luteolin** ***C*-hex (Lut** ***C*-hex)** probably (iso)orientin	C_21_H_20_O_11_	9.51	**447.094 [M-H]**^−^	→28.7 eV: 429 [E_1_]^−^, 411 [E_2_]^−^, **357 [**^**0,3**^**X]**^−^, 339 [^0,3^X-H_2_O]^−^, **327 [**^**0,2**^**X]**^−^, 297 [^0,1^X]^−^, 285 [Y_0_]^−^
	**10**	**luteolin** ***C*-hex**, ***C*-pen (Lut** ***C*-hex** ***C*-pen)**	C_26_H_28_O_15_	8.54	**579.136 [M-H]**^−^	→31.6 eV: 519 [(M-H)−60]^−^, **489 [(M-H)−90]**^−^, 459 [(M-H)−120]^−^, 429 [(M-H)−150]^−^, 399 [Ag+113]^−^, 369 [Ag+83]^−^
	**11**	**luteolin** ***C*-hex, 2”-*O*-dhex (Lut** ***C*-hex** ***O*-dhex)**	C_27_H_30_O_15_	9.74	**593.152 [M-H]**	→31.9 eV: 575 [(M-H)−18]^−^, 503 [(M-H)−90]^−^, **473 [(M-H)−120]**^−^, **429 [(M-H)−164]**^−^, 357 [Ag+71]^−^, 339 [Ag+71-18]^−^, 327 [Ag+41]^−^, 309 [Ag+41-18]^−^
	**12**	**chrysoeriol** ***C*-hex (Chr** ***C*-hex)** probably (iso)scoparin	C_22_H_22_O_11_	12.10	**461.110 [M-H]**^−^	→29.0 eV: 443 [E_1_]^−^, 371 [^0,3^X]^−^, 353 [^0,3^X-H_2_O]^−^, **341 [**^**0,2**^**X]**^−^, 298 [Y_0_-H]^−^
	**13**	**chrysoeriol** ***C*-hex**, ***C*-pen (Chr** ***C*-hex** ***C*-pen)**	C_27_H_30_O_15_	10.40	**593.152 [M-H]**^−^	→31.9 eV: 575 [(M-H)−18]^−^, 533 [(M-H)−60]^−^, **503 [(M-H)−90]**^−^, **473 [(M-H)−120]**^−^, 443[(M-H)−150]^−^, 413 [Ag+113]^−^, 383 [Ag+83]^−^
	**14**	**chrysoeriol** ***C*-hex, 2”-*O*-dhex (Chr** ***C*-hex** ***O*-dhex)**	C_28_H_32_O_15_	12.02	**607.168 [M-H]**^−^	→32.1 eV: 487 [(M-H)−120]^−^, **443 [(M-H)−164]**^−^, 371 [Ag+71]^−^, 353 [Ag+71-18]^−^, 341 [Ag+41]^−^, **323 [Ag** + **41-18]**^−^
	**15**	**tricin di-*O*-hex (Tri di-*O*-hex)**	C_29_H_34_O_17_	12.54	**653.173 [M-H]**^−^	→33.1 eV: **329 [Y**_**0**_**]**^−^
	**16**	**tricin** ***O*-hex**, ***O*-dhex (Tri** ***O*-hex** ***O*-dhex)**	C_29_H_34_O_16_	14.02	**637.178 [M-H]**^−^	→32.7 eV: **329 [Y**_**0**_**]**^−^
Others	**17**	sinapic acid (SinAc)*	C_11_H_12_O_5_	10.46	**223.061 [M-H]**^−^	→23.0 eV: 208 [C_10_H_8_O_5_]^−^, **193 [C**_**9**_**H**_**5**_**O**_**5**_**]**^−^, 164 [C_9_H_8_O_3_]^−^, 149 [C_8_H_5_O_3_]^−^, 135 [C_8_H_7_O_2_]^−^, 121 [C_7_H_5_O_2_]^−^, 93 [C_6_H_5_O]^−^
	**18**	salicylic acid glucoside (SAG)*	C_13_H_16_O_8_	5.29	**299.078 [M-H]**^−^	→25.0 eV: **137 [(M-H)-hex]**^−^, 93 [C_6_H_5_O]^−^
	**19**	**aromatic compound (Arom)**	C_11_H_10_O_6_	11.70	**237.041 [M-H]**^−^	→23.4 eV: **121 [C**_**7**_**H**_**5**_**O**_**2**_**]**^−^, 115 [C_4_H_3_O_4_]^−^, 77 [C_6_H_5_]^−^

Metabolites that were validated using a reference standard are marked with an asterisk. Abbreviations for metabolites used in other parts of the manuscript are shown in brackets. For each metabolite, dominant ions in MS and MS/MS (fragments of the dominant ion in MS mode, collision energy indicated) mode are shown, with the dominant ions indicated in bold. For fragments, sum formulas or (for flavonoid glycosides) characteristic diagnostic ion types are given. Due to in-source fragmentation, the [M-H]^−^ ion of DIMBOA was nearly absent in MS mode and the MS spectrum was dominated by the fragment 164 m/z; thus, the latter ion was used for further fragmentation and quantification of this metabolite. Ag, aglycone; BXD, benzoxazinoid; dhex, deoxyhexosyl; hex, hexosyl; pen, pentosyl.

## Discussion

This study revealed that deficit irrigation affects wheat growth and WUE_apl_ as well as the (semi-)polar metabolome of flag leaves, providing thus novel insights into the importance of irrigation frequency next to irrigation volume. A reduction in F_v_/F_m_ indicates that plants are stressed^3^, but F_v_/F_m_ is usually only responsive under severe drought stress^33^ and especially in drought-sensitive genotypes^34^. In our study, no significant changes in F_v_/F_m_ were found in drought-stressed plants 5 h after watering all plants, suggesting that the photosystem II complexes were not chronically damaged under our deficit irrigation treatments and that the effects of drought on the different plant parameters measured were mediated by other factors, as discussed below.

Our first hypothesis, which predicted a decreased aboveground dry mass under deficit irrigation, especially at pd, is partly supported by our results. Drought-stressed plants produced much less aboveground vegetative biomass than control plants. Similarly, a lower aboveground biomass under drought has been observed in most plant species both under laboratory and field conditions^6,35^. These findings may be due to a lower stomatal conductance and CO_2_ assimilation rate under drought^11,36^. A lower nutrient mobility within the soil, nutrient uptake by roots and translocation of nutrients to shoots^37^ under water shortage might also play a role. Plant responses to drought and the coordination of growth-related processes under drought are regulated by complex signalling networks, involving abscisic acid^38,39^. Relative to shoot biomass, drought stress-exposed wheat plants may have invested more in root growth and/or adjusted the root architecture, which are common responses to water deficiency to optimise water and nutrient uptake from the soil^40,41^. The findings that the generative biomass at T2 was not affected by the drought treatments and that the harvest index was increased under drought indicate that the plants allocated relatively more resources into generative plant parts when suffering from drought. However, in contrast to our prediction, the aboveground vegetative and generative biomass were not or only slightly lower in pd compared to cd plants. In contrast, wheat plants that were watered to field capacity with different irrigation frequencies showed a reduced shoot dry mass with decreasing irrigation frequencies^42^. In the field, highest grain yields for winter wheat were achieved at medium watering frequency^43^. In addition to different effects of drought and irrigation frequencies on grain yield, the quality of the produced grains may be affected by drought and this aspect should be addressed in future studies.

Secondly, as we had assumed, WUE_apl_ was significantly higher in drought-exposed compared to control plants and the difference was more pronounced for cd than for pd plants. The fact that stomatal closure under drought can restrict transpiration before it inhibits photosynthesis can explain why WUE_i_ is often higher under (mild) drought^8^. This physiological response may partly explain the higher WUE_apl_ under drought found in the present study. Furthermore, plant growth rates under these conditions have to be considered, i. e., the capabilities of the plants to use the ongoing photosynthesis for biomass production. Additionally, lower evaporation from dry soils^44^ and root systems that take up water more effectively (see above) probably contribute to the higher WUE_apl_ in drought-stressed plants. For several temperate grassland species WUE_i_, estimated *via* foliar δ^13^C, was lower in pulsed-watered compared to regularly-watered grasses under water deficit conditions, but did not differ in dicot species^45^. Moreover, WUE_apl_ was similarly increased in vegetative and generative parts of drought-stressed plants in the present experiment. Likewise, in two other wheat cultivars the WUE for biomass yield as well as that for grain yield were significantly higher in drought-stressed compared to well-watered plants^46^. Finally, in the present study, the WUE_apl_ was lower in pd than in cd plants. This finding could be due to higher evaporation from soil after pulse irrigation and/or differences in root systems between cd and pd plants. In a previous experiment in which wheat plants of the same cultivar were differently irrigated and harvested after fruit ripening, the WUE_apl_ based on the grain yield, but not that based on the vegetative biomass, was higher in cd than in pd plants^47^. Both studies highlight that the investment of wheat plants into vegetative and generative growth under drought depends on the irrigation frequency and the improvement of the WUE_apl_ and that the harvest index is limited at low irrigation frequencies. For cereal agriculture in water-limited systems a low WUE_apl_ is unfavourable, because it implies that rain or water delivered artificially are not effectively used for plant biomass and yield production. Thus, under future climate change scenarios, selecting for a high WUE_apl_ might become more important.

The phytometabolomes differed pronouncedly between flag leaves harvested at the two time points and in particular between leaves of ctr plants and pd plants. We predicted that the flag leaf metabolome responds to drought treatments with an increase in the concentrations of many metabolites. Indeed, more features were increased in pool size than decreased. Some of these metabolites increased in pool size might be involved in osmoregulation^10^ or in plant responses to secondary oxidative stress^48,49^, whereas others probably are plant defence compounds. In various plant species, concentrations and contents of specialised metabolites were shown to be higher under drought^15,16^. Somewhat less metabolites decreased in their pool sizes in our wheat plants under drought. This might be due to a lower photosynthetic activity^50^ and/or reduced uptake of N, P and K^51^ under drought. Both probably limit metabolic processes and intensify trade-offs in investment of resources into growth *versus* specialised metabolism. Next to potential trade-offs, the simultaneous increases and decreases in pool sizes of different metabolites provide evidence that large parts of the metabolic changes found here in wheat flag leaves are not simply a concentration effect caused by lower biomass of drought-stressed plants. In contrast to our findings, in needles of Scots pine more metabolic features including specialised metabolites were decreased than increased under severe drought^18^, indicating that plant responses to drought may be species-specific and depend on many factors.

We found several common metabolic responses of the flag leaves to both drought treatments at each of the two time points, indicating that some metabolites already respond at a low stress threshold and generally respond to a reduced water supply, regardless of the irrigation frequency. Some features were commonly modulated throughout time, whereas others were exclusively responsive at one time point. At T2, a higher proportion of features was commonly modulated in flag leaves of both drought treatments than at T1. These findings highlight that the duration of exposure to drought stress plays an important role for the extent of metabolic changes, as also shown for other plant species^16,18^. Furthermore, at the two harvest time points plants were in different developmental stages, in which flag leaves might have distinct functions and might therefore respond differently to drought. However, at both time points the majority of metabolic features was specifically modulated by only one of the drought treatments, meaning that the cd and pd treatments had distinct effects on the flag leaf metabolome. In line with our assumption, these metabolic responses were more pronounced in flag leaves at a lower irrigation frequency (pd) compared to the cd treatment.

In line with other studies^28,31,32,49^, we detected several metabolites derived from the shikimic acid pathway in wheat leaves, including salicylic acid glucoside, a BXD and BXD glucosides as well as flavonoid glycosides. The common decrease of salicylic acid glucoside under both drought treatments at both time points may be due to a release of salicylic acid, which mediates plant responses to abiotic stress including drought^52,53^. BXD aglyca have been found to be enhanced under drought stress in maize^54^ and wheat^28^ seedlings. These studies did not investigate BXD glucosides, although BXDs mainly occur as glucosides in plants^55^. We could not detect any drought effect on DIMBOA, whereas DIMBOA glucoside and HDMBOA glucoside were increased in pool size in flag leaves in pd plants at the first time point. Both BXDs and BXD glucosides are inducible by herbivore feeding^56^ and have insecticidal properties^25,49^. An increase of BXDs and their glucosides in wheat leaves under pulsed drought might thus be one beneficial aspect of climate change for agriculture, because these metabolites mediate a higher resistance against herbivores. Furthermore, these metabolites may accumulate in grains and affect human nutrition, because they are thought to have health-promoting effects^57^.

Some glycosylated flavones were lower and others higher in drought-stressed compared to control plants. *C*-glycosyl flavones are prominent compounds in wheat that are affected by abiotic stress^31,32,58^. Furthermore, *C*-glycosyl flavones can serve as indicators of the nitrogen status of a plant^31^ and a reduction in soil moisture can lead to nutrient limitation in plants. Thus, the shifts in flavone glycoside metabolism found in the present experiment may be related to plant nutrition. Moreover, flavonoids in general have antioxidant properties^30^. Hence, the shifts in flavonoid metabolism observed here may also be related to mitigation of secondary oxidative stress. For breeding of drought-tolerant wheat cultivars, it will be important to understand the links between drought-induced changes in flavonoid concentrations and the expression levels of the genes encoding the corresponding biosynthetic enzymes^58^.

In conclusion, not only the volume of irrigation but also its frequency and the duration of deficit irrigation modify plant growth, WUE and the phytometabolome, including metabolites derived from the shikimic acid pathway. These findings are especially important for irrigation scheduling of crops in the context of climate change. Studies on drought effects should consider plant responses at different time points and incorporate dynamics of soil and plant water status to link these parameters, F_v_/F_m_, WUE_apl_ and the plant metabolome. Future transcriptomics and proteomics studies targeting the genes and proteins involved in the drought-responsive metabolic pathways may help to understand genetic control and regulation of metabolic processes under drought. Moreover, potential priming effects due to previous drought events that alleviate negative effects of drought during grain development^59^ should be considered. In combination with drought, other factors of climate change such as heat waves, which are predicted to increase in frequency and intensity, might have more detrimental impacts than if these stresses occur individually^60^. In line with the leaf metabolome, volatile organic compounds may be affected by climate change-associated stress^11,61^. In this study, we demonstrate how a wheat cultivar with medium drought tolerance is affected by irrigation frequency under drought. Under field conditions, the system is more complex and plant responses will need to be examined in the context of various abiotic and biotic stress factors. Especially, the effects of irrigation frequency under drought should be investigated for cultivars differing in abiotic stress tolerance. Together with results of further studies, it might be possible to determine optimal irrigation strategies in the field for particular conditions.

## Materials and Methods

### Plant cultivation, drought treatments and harvest

The experiment was performed from December 2016 to May 2017. Seeds of spring wheat (*T. aestivum* cv. Tybalt, von Borries-Eckendorf, Leopoldshöhe, Germany) were germinated in a steamed (90 °C, 8 h) 1:1 mixture of river sand and soil (Fruhstorfer Pikiererde, Hawita Group, Vechta, Germany) in a greenhouse at 22 °C, 46% relative humidity (r.h.) and a photoperiod of 12 h:12 h light:dark (L:D). Six days after sowing, five seedlings were planted together in an equal distance of 6 cm surrounding a central seedling in each of 60 pots (4 L, 15.7 × 15.7 × 23.3 cm; Meyer, Rellingen, Germany) to simulate natural competition. Each pot contained 4,185 g of the substrate mentioned above, with a substrate water content of 23% (determined gravimetrically in a previous experiment^47^). Pots had holes at the bases to allow draining and were placed on dishes to prevent water loss after leaching. Henceforward, the greenhouse settings were 11 °C at 75% r.h. and a photoperiod of 12 h:12 h L:D. Ambient light was supported by 400 W HPI-T Plus lamps (Philips, Amsterdam, Netherlands) and pot positions were initially randomly changed twice a week, from the start of the irrigation treatments onwards once a week. From five to six weeks after sowing, the temperature was increased stepwise to 14 °C (at 64% r.h.) and then to 19 °C (at 58% r.h.) and the photoperiod changed to 14 h:10 h L:D and then to 16 h:8 h L:D. After seedlings were transferred to the pots, the substrate water content was determined gravimetrically for 15 randomly chosen pots every other day, weights were averaged, and the volume of tap water needed to reach a soil water content of 23% was added to each pot. Forty six days after sowing, when stem elongation started (BBCH stage: 30^62^), pots were randomly assigned to one of three irrigation treatments with 20 pots per group: a well-watered control (ctr), continuously drought-stressed (cd) and pulsed drought-stressed (pd) plants (Fig. 1). Pots of the ctr treatment were adjusted to a substrate water content of 23% every other day. Pots of the drought treatments were not watered until they reached a mean gravimetric soil water content of 11% (measured from 15 randomly chosen pots that were weighed), which occurred 60 days after sowing. From 62 days after sowing onwards, cd plants were provided with 40% of the water amount that ctr plants received and watered in that way every other day. For plants of the pd treatment, the cumulative amount of water given to cd plants within 8 days (4 watering events) was provided only every 8 days, starting at 68 days after sowing (Fig. 1). All water was retained within the pots and surrounding dishes, but evaporation from the substrate surface and the dishes was not restricted. All plants were fertilised with 5 g and 3 g of fertiliser (Plantosan N-P-K 20-10-15, containing 6% Mg, 2% S, traces of B, Cu, Fe, Mn, Mo, Zn; Manna, Düsseldorf, Germany) at 32 and 68 days after sowing, respectively, directly before watering all pots.

On day 76 and 92 after sowing (30 and 46 after the start of the irrigation treatments), the maximum quantum yield of photosystem II (F_v_/F_m_) was determined 5 h after watering all plants. This parameter was measured at the flag leaf of the central plant of each pot after 15 min of dark adaptation with a leaf clip using a Mini-PAM photosynthesis yield analyser (Walz GmbH, Effeltrich, Germany). Seventy seven days after sowing (T1; 31 days after start of irrigation treatments), plant heading started and first spikelets were just visible (BBCH stage: 51). At this time point the flag leaf of the main shoot was harvested from one randomly chosen surrounding plant of each pot (*n* = 10 per treatment) for subsequent metabolomics analysis. Flag leaves were harvested in the same way from plants of 10 other pots per treatment at a later plant age, i.e., 93 days after sowing (T2; 47 days after start of irrigation treatments), when plants had developed full inflorescences (BBCH stage: 59). The two harvests were performed one day after plants of all treatments had been watered. After harvest, flag leaves were immediately frozen in liquid nitrogen and stored at −80 °C. The remaining aboveground plant parts were harvested from each pot and dried at 40 °C for 96 h. For T1, the total aboveground dry mass of all plants within one pot (lacking the one flag leaf harvested for metabolic fingerprinting) and the amount of water provided until the first harvest for each pot were used to calculate the applied water use efficiency (WUE_apl_) as ratio of aboveground dry mass to the water amount provided, following Boyle *et al*.^5^. For T2, the aboveground dry mass of vegetative (stems and leaves) and generative parts (ears) was determined separately and the harvest index calculated as the ratio of grain yield to total aboveground dry biomass. Furthermore, vegetative and generative WUE_apl_ were calculated using the total amount of water provided over the entire experimental period.

### Metabolic fingerprinting of flag leaves

Metabolic fingerprinting of flag leaves was performed following Hanhineva *et al*.^63^ with some modifications. For analysis of the (semi-)polar foliar metabolome, 4 cm long pieces were cut from the centre of each leaf blade and weighed. Leaf pieces were milled in 300 µL of 75% cold methanol (Th. Geyer GmbH & Co KG, Renningen, Germany) with 0.1% formic acid (FA; p.a., eluent additive for LC-MS, ~98%, Sigma-Aldrich, Steinheim, Germany), containing hydrocortisone (>98%; Sigma-Aldrich) as internal standard. Samples were sonicated for 15 min and centrifuged for 5 min. Supernatants were filtered (0.2 µm syringe filters, Phenomenex, Torrance, USA) and analysed by ultra-high performance liquid chromatography coupled to quadrupole time-of-flight mass spectrometry (UHPLC-QTOF-MS/MS; UHPLC: DionexUltiMate 3000, Thermo Fisher Scientific, San José, USA; QTOF-MS/MS: compact, Bruker Daltonics, Bremen, Germany). In addition, four blank samples were measured. All samples of the different treatment groups as well as the blanks were injected within one continuous batch in alternating order. Metabolites were separated on an UPLC BEH C18 column (Waters Acquity, 100 × 2.1 mm, 1.7 µm particle size, with guard column; Waters GmbH, Eschborn, Germany) at 35 °C and a flow rate of 0.3 mL min^−1^. A gradient from eluent A [5:95 acetonitrile:Millipore-H_2_O (v:v) with 0.1% FA; acetonitrile: LC-MS grade, Fisher Scientific, Loughborough, UK] to eluent B (acetonitrile with 0.1% FA) with the following steps was used: 0–28% B within 22 min, 28–40% B within 0.5 min, 40–100% B within 0.5 min, hold for 1.5 min. Metabolites were subjected to negative electrospray ionisation and line spectra recorded for the mass-to-charge (m/z) range 50–1300 at 8 Hz with the following settings: end plate offset 500 V, capillary voltage 3,000 V, nebuliser (N_2_) pressure 3 bar, dry gas (N_2_, 275 °C) flow 12 mL min^−1^, quadrupole ion energy 4 eV, low mass 90 m/z and MS collision energy 7 eV. MS/MS spectra (50–1300 m/z) were acquired with N_2_ as collision gas in AutoMSMS mode. A Na(HCOO)-based calibration solution was measured preceding each sample. To assist metabolite identification, some samples were additionally analysed using a longer chromatographic gradient, lower spectra rates (2 Hz), positive electrospray ionisation (capillary voltage 4,500 V) and multiple reaction monitoring to assess specific fragmentation patterns. Moreover, several reference standards were measured using the same methods.

### Data processing and statistical analyses

All statistical analyses were performed in R^64^ (versions 3.4.2 and 3.6.2). F_v_/F_m_ data were analysed using a generalised linear model with Gamma-distributed errors and an inverse link function including the factors ‘irrigation treatment’ (factor levels: ctr, cd, pd) and ‘time point’ (factor levels: T1, T2) and their interaction term. The total aboveground dry mass and WUE_apl_ for T1, the vegetative and generative aboveground dry mass and WUE_apl_ for T2 as well as the harvest index were analysed with linear models including the factor ‘irrigation treatment’. Model assumptions (normality and homoscedasticity of residuals) were checked using diagnostic plots and Shapiro-Wilk tests. If overall effects were significant, manual contrasts were calculated for selected comparisons (ctr *vs*. cd and cd *vs*. pd) within each model using the R package *contrast*. *P* values for contrasts were corrected according to Holm within each model.

For chromatogram processing, mass axis recalibration based on the Na(HCOO) m/z cluster and peak picking using the Find Molecular Features algorithm were performed in Compass DataAnalysis 4.4 (Bruker Daltonics). The following settings were used: signal-to-noise threshold 3, correlation coefficient threshold 0.75, minimum compound length 28 spectra, smoothing width 6, allowing common adducts and neutral losses for bucket generation. Buckets were aligned across samples using Compass ProfileAnalysis 2.3 (Bruker Daltonics) using the m/z with the highest intensity as bucketing basis and allowing deviations of 0.2 min RT (retention time) and 6 mDa (m/z), respectively. The intensities of the resulting metabolic features (each described by a RT and a m/z value) were related to the intensities of the [M + HCOOH-H]^−^ ion of the internal standard hydrocortisone. Only those features were retained in the dataset, whose mean intensities in at least one treatment group (irrigation treatment *x* time point) were at least 50 times higher than their mean intensity in the blanks. Furthermore, features had to occur in at least half of the replicates of at least one treatment group. Feature intensities were divided by the sample fresh weights.

A PCA including all metabolic features that were retained (see above) was performed after autoscaling (i. e., mean-centering and scaling to unit variance) and replacing zeros by small random numbers (10^−13^ to 10^−12^). For selected pairwise group comparisons (drought stress groups against control group within each time point), volcano plots were generated in R based on (log_2_-scaled) fold changes (i.e., mean feature intensities in drought stress group divided by mean intensities in control group) and (-log_10_-scaled) *P* values derived from two-sided Mann-Whitney *U*-tests (calculated in R). For features, which were absent in one of the two groups, fold changes were set to the minimum (present only in control group) or maximum (exclusively in drought stress group) fold changes observed in the group pair, respectively. Features were considered as being modulated by drought stress, if the fold change was <0.5 (<−1 on log_2_ scale; lower intensity in drought stress group) or >2 (>1 on log_2_ scale; higher intensity in drought stress group) and their intensities significantly (unadjusted *p* < 0.05) differed between the groups. *P* values were corrected for multiple testing according to Benjamini and Hochberg (1995)^65^ using a false discovery rate of 0.05. To visualise the overlap of features modulated by the different drought stress treatments (cd, pd) and at different time points (T1, T2), Venn diagrams (function *venn* in R package *gplots*) were plotted.

For the identification of metabolites, only metabolic features occurring at RT > 0.3 min were considered, as many primary metabolites co-elute earlier and this study focuses on specialised metabolites. The annotation tools of MetaboScape 2.0 and Data Analysis 4.4 (Bruker Daltonics) were used. With Smart Formula 3D and/or MetFrag^66^, molecular formulas of parent ions and fragments were generated based on their m/z and isotopic pattern fit. Using MetFrag, *in-silico* fragmentation based on compounds listed in PubChem as well as spectral matching against entries in the MassBank of North America (https://mona.fiehnlab.ucdavis.edu/) were used to derive structure suggestions. RT, UV/VIS spectra, dominant ion types and m/z (MS and MS/MS spectra) were compared to those of reference standards listed in spectral libraries associated with the vendor software (Bruker Daltonics) and those listed in an in-house database. Identifications of BXDs and their glucosides were further validated by diagnostic ions (MS and MS/MS)^56^. *C*/*O*-glycosyl flavones, which occur in wheat but for which no reference standards were available, were putatively identified based on distinct published fragmentation patterns^67,68^. In general, metabolite identifications were done using negative electrospray data, but MS and MS/MS spectra were also checked for positive electrospray data for some samples. A pathway map including all identified metabolites was constructed based on the KEGG database^69^, including additional specific information for the biosynthesis of BXDs^70^. All figures were further edited in Corel Draw Graphics Suite X5 (Corel Corporation, Ottawa, Canada, 2010).

## Data Availability

The bucket table with metabolic features will be deposited on the MetaboLights platform (http://www.ebi.ac.uk/metabolights) upon acceptance of the manuscript. All other datasets generated during the current study are available from the corresponding author on reasonable request.
